# Severe asymptomatic coronary obstruction in chronic hemodialysed patient – a case report

**Published:** 2016

**Authors:** C Voiculeț, O Zara, I Văcăroiu, C Bogeanu, T Tiron, F Turcu, G Aron, A Ciocâlteu

**Affiliations:** * Department of Internal Medicine, “Sf. Ioan” Clinical Emergency Hospital Bucharest, Romania; ** Department of Angiography, Catheterization and Electrophysiology, “Sf. Ioan” Clinical Emergency Hospital Bucharest, Romania; *** Department of Nephrology and Dialysis, “Sf. Ioan” Clinical Emergency Hospital Bucharest, Romania; **** 3rd Clinical Department, “Carol Davila” University of Medicine and Pharmacy Bucharest, Romania; ***** 1st Clinical Department, “Carol Davila” University of Medicine and Pharmacy Bucharest, Romania

**Keywords:** hemodialysis, vascular calcification, asymptomatic coronary artery disease, angiography

## Abstract

**Introduction.** Arterial stiffness and vascular calcifications are independent predictors of cardiovascular morbidity and mortality in the chronic kidney disease (CKD) stage 5D population. According to the guidelines, patients on renal replacement therapy represent a very high cardiovascular risk class.

**Case report.** We report the case of a 67-year-old hypertensive male patient, known with CKD stage 5D on hemodialysis (three times per week), secondary bone mineral disease, admitted for progressive right leg pain. The physical examination detected right dorsalis pedis artery pulse absence. Blood biochemistry emphasized hypercalcemia, hyperphosphatemia, increased alkaline phosphatase, metabolic acidosis, hypoalbuminemia, iPTH values above upper limits. The X-ray of right shin highlighted a vascular calcification with a “train track” aspect on the tibial-peroneal artery trunk and the thoracic X-ray (performed with low ray regime) showed calcium deposits in coronary arteries walls. Legs arteriography and coronary angiography were performed revealing multiple lesions on investigated vessels with an 80% narrowing of right coronary artery. The particularity of the case lies in the absence of angina in a chronic hemodialysis patient in whom multiple significant angiographically stenosis of the coronary arteries were found and successful endovascular therapy was performed.

**Conclusion.** The broadening of the indication for coronary angiography should be considered in certain asymptomatic CKD stage 5D patients based on a risk score involving calcium, phosphate, PTH and acid-base imbalances, while considering their major influence on the structure and tone of vascular walls thus on cardiovascular morbidity and mortality rates.

**Abbreviations.** ABI = ankle-brachial index,CAD = coronary artery disease,CKD = chronic kidney disease,CT = computed tomography, EBCT = electron-beam computed tomography,ESRD = end-stage renal disease,GFR = glomerular filtration rate,iPTH = intact parathormon,PCI = percutaneous coronary intervention

## Introduction

Cardiovascular disease is the major cause of high mortality not only in chronic kidney disease (CKD) patients but also in the general population. In this category of patients, the disturbance of calcium-phosphate homeostasis confers an increased risk for vascular calcification, which is one of the powerful predictors of cardiovascular morbidity. Therefore, screening for coronary artery disease (CAD) in the dialysis patients is very important, but, until now, there has been no consensus established regarding the screening methods [**1**-**5**].

Asymptomatic coronary artery disease is very common in the dialysis population and the absence of symptoms cannot rule out coronary lesions because it is secondary to autonomic neuropathy and a decreased tolerance to effort rather than to hemodynamic changes. Coronary angiography remains “the gold standard” for the diagnosis of CAD, in most cases,also providing the appropriate therapy during the same procedure [**6**].

## Case report

A 67-year-old male patient was referred to our Clinical Emergency Hospital, to the Department of Internal Medicine, for pain in the right leg when walking 50 meters, symptoms onset two months before, progressively increasing. The previous medical history included CKD stage 5D and thrice-weekly conventional hemodialysis for over 6 years, high BP values treated with calcium channel blocker, bone mineral disease secondary to CKD treated with calcium (500 mg/day), Alphacalcidol (0.25 µg/day) and calcium carbonate as a phosphate binder (9 tablets/day).

The clinical examination revealed discrete mucocutaneous pallor, the presence of left brachiobasilic arteriovenous fistula, blood pressure 140/90 mmHg, regular pulse rate 78 beats/minute, right dorsalis pedis artery pulse absence and anuria.

Blood biochemistry showed discrete normochromic and normocytic anemia, high values for urea and creatinine, hypercalcemia, hyperphosphatemia, increased alkaline phosphatase, metabolic acidosis, hypoalbuminemia, iPTH (intact parathormon) above upper limits (**Table 1**).

**Table 1:** Laboratory tests

Biological constant	Patient’s values	Laboratory normal range
Hemoglobin (g/ dL)	12.1	14 – 17
Creatinine (mg/ dL)	6.3	0.83 – 1.24
Urea (mg/ dL)	103	10 – 50
Calcium (mg/ dL)	10.5	8.5 – 9.5
Phosphate (mg/ dL)	7.1	3.5 – 5.5
Alkaline phosphatase (UI/ L)	27	< 20
Serum bicarbonate (mEq/ L)	17.5	20 – 22
Albumin (g/ dL)	3.3	> 4
iPTH (pg/ mL)	1173	150 – 300

**Fig. 1:**
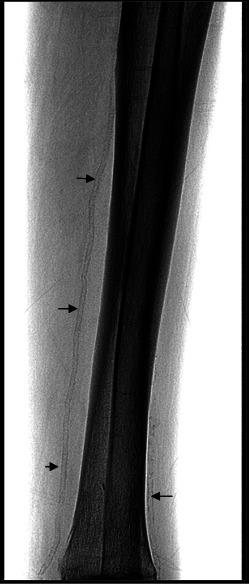
Vascular calcification with a “train track” aspect on the right anterior and posterior tibial artery walls

Right leg X-ray revealed calcium deposits with a “train track” aspect on the right anterior and posterior tibial artery walls (**Fig. 1**).

**Fig. 2:**
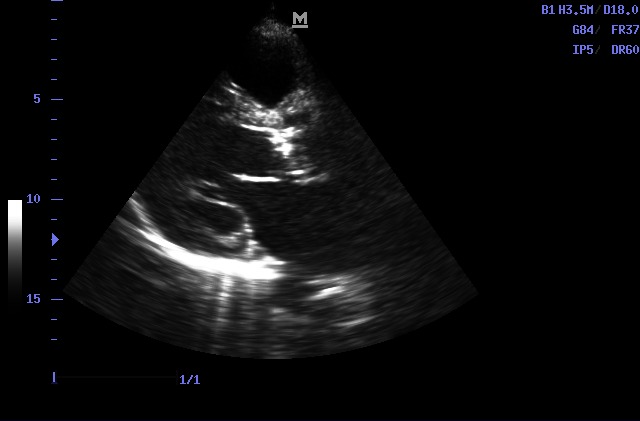
Aortic valvular calcification

**Fig. 3:**
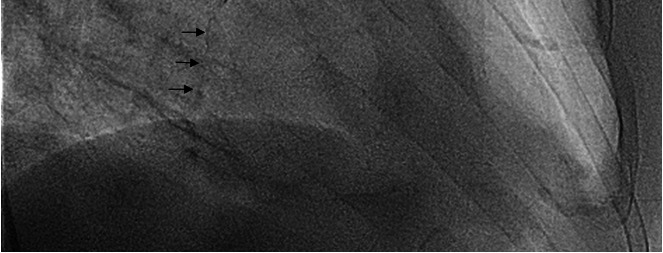
Right coronary artery – vascular calcification on plain radiography

The patient was further evaluated by determining the ankle-brachial index (ABI), arterial Doppler conducting legs, chest X-ray, and echocardiography. Right ABI was 0.7 and left ABI was 0.8. Doppler ultrasonography showed lower limbs arterial atheromatous and diffuse bilateral calcifications with no flow to the distal segment of the right posterior tibial artery. Echocardiography revealed aortic valve calcification and chest X-rays determined diffuse calcium deposits located in the epicardial coronary arteries (**Fig. 2**,**3**).

**Fig. 4:**
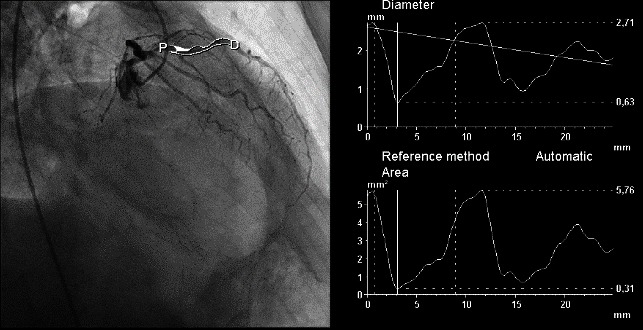
Significant stenosis of interventricular anterior coronary

**Fig. 5:**
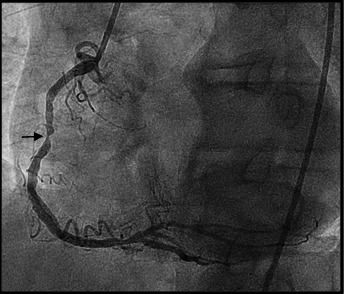
Right coronary artery with significant stenosis in second segment

**Fig. 6:**
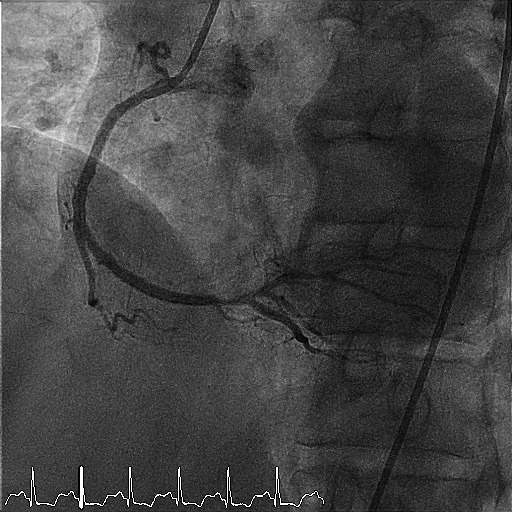
Right coronary artery after angioplasty

**Fig. 7:**
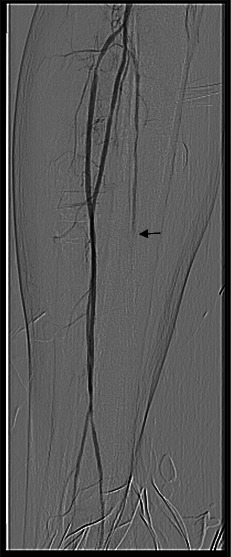
Posterior tibial artery occlusion in middle segment

The arteriography was established based on clinical and laboratory data, the indication for both coronary angiography and lower limbs. Coronary angiography revealed a diffuse vascular calcification, long interventricular artery stenosis in the second segment of 70–80%, and 80% stenosis in the medium segment of the right coronary artery, therefore angioplasty with stent implantation was performed (**Fig. 4**-**6**). Lower limbs angiography showed a calcification throughout the arterial axis with 80% stenosis in the proximal segment of the anterior tibial artery and an occlusion in the middle segment of the posterior tibial artery, bilaterally (**Fig. 7**).

The particularity of the case lies in the presence of pain in the right leg but the absence of angina in a patient with CKD stage 5D on dialysis who had an angiographic occlusion of the middle segment of right posterior tibial artery and a significant right anterior tibial artery stenosis, and who required coronary artery percutaneous intervention for significant right coronary artery stenosis.

## Discussions

Cardiovascular disease accounts for approximately 50% of the deaths among patients undergoing chronic dialysis [**7**-**14**]. Once the glomerular filtration rate (GFR) decreases, a significant prevalence of vascular calcification is observed (consequently vascular/valvular calcification) much sooner in CKD individuals than in the general population [**1**,**10**,**14**]. A 30% decrease in GFR is associated with a 20–30% increased risk of major cardiovascular events and all-cause mortality in uremic patients [**15**-**17**].

In patients with end-stage renal disease (ESRD), the dysregulation of calcium and phosphate metabolism is the main factor involved in the development of vascular calcifications. Hypercalcemia and increased phosphate have direct effects on the vascular smooth muscle cell, which stimulate osteogenic trans-differentiation, vesicle release, apoptosis, loss of inhibitors and matrix degradation to drive arterial-wall mineralization. Different studies concluded that the imbalance responsible for osteoblast-like phenotype formation can induce the development and also the progression of vascular calcification [**1**,**18**,**19**].

Braun et al. concluded that coronary-artery calcification was much more common among adult patients with ESDR than among normal subjects of the same age and sex [**20**]. Oh et al.reported that coronary artery calcification has been shown in up to 92% of young CKD hemodialysis patients with computed tomography (CT) scan [**21**]. In contrast, Sharplesshowed that coronary artery calcification measured by electron-beam computed tomography (EBCT) is not an accurate marker of the degree of vessel stenosis in CAD in CKD patients and should not be used as a single screening test for atherosclerotic coronary disease [**22**]. De Vriesesuggested that coronary angiography is the best method to diagnose CAD. The rational use of coronary angiography as a screening test implies that the finding of significant stenosis is followed by a percutaneous intervention [**6**].

Joki et al.noted a prevalence of significant coronary artery disease (stenosis of at least 75%) in 54% of the asymptomatic patients with ESRD, examined within one month of initiation of hemodialysis [**23**]. Charytan et al. performed coronary angiography in 67 asymptomatic hemodialysis patients and they found coronary artery stenosis of at least 50% in 42% of the patients, 75% of whom had multi-vessel involvement [**24**].

Yasuda et al. performed coronary angiography in 259 hemodialysis patients. The patients who had significant lesions were informed about the benefits and risks of percutaneous coronary intervention (PCI). It has been observed that both all-cause and cardiac 5-year survival rates were strongly higher in the PCI group than in the medication-only group [**6**,**25**].

The life span of ESRD patients is reduced due to cardiovascular diseases. Hence, the early diagnosis of vascular calcifications increases the survival rate in patients with CKD.

## Conclusion

The clinical and biological elements that reveal severe mineral bone disease associated with CKD stage 5D should be considered as criteria to expand indications for angiography in the absence of angina, as these patients usually avoid intense and prolonged physical effort. Furthermore, bone mineral disorders have a major impact on the morphology and vascular walls tone in these patients, thus culminating with increased cardiovascular morbidity and mortality 
